# A mixed methods descriptive study of a diverse cohort of African American/Black and Latine young and emerging adults living with HIV: Sociodemographic, background, and contextual factors

**DOI:** 10.1186/s12889-025-21869-3

**Published:** 2025-02-14

**Authors:** Marya Gwadz, Leo Wilton, Charles M. Cleland, Samantha Serrano, Dawa Sherpa, Maria Fernanda Zaldivar, Robin Freeman, Stephanie Campos, Nisha Beharie, Corey Rosmarin-DeStefano, Prema Filippone, Michelle R. Munson

**Affiliations:** 1 Silver School of Social Work, New York University, 1 Washington Square North, New York, NY 10003 USA; 2 Department of Human Development, State University of New York at Binghamton, 4400 Vestal Parkway East, Binghamton, NY 13902 USA; 3 Faculty of Humanities, University of Johannesburg, PO Box 524, Auckland Park, Johannesburg, 2006 South Africa; 4 Department of Population Health, New York University Grossman School of Medicine, NYU Langone Health, 180 Madison Avenue, 2-53, New York, NY 10016 USA; 5 Department of Public Health, School of Health Science and Practice (SHSP), New York Medical College, 30 Plaza West, Room 223, Valhalla, NY 10595 USA; 6 North Jersey Community Research Initiative, 393 Central Avenue, Newark, NJ 07103 USA

**Keywords:** HIV care continuum, HIV viral non-suppression, Mixed methods, Young adult, Emerging adults, Black, Latino/Latine, Immigration, Contextual factors, Social action theory

## Abstract

**Background:**

American/Black and Latine (AABL) young/emerging adults living with HIV in the United States (US) have consistently failed to meet targets for HIV care/medication engagement. Among this population, those with non-suppressed HIV viral load are understudied, along with immigrants and those with serious socioeconomic deprivation. Guided by social action theory, we took a mixed methods approach (sequential explanatory design) to describe sociodemographic, background, and contextual factors, and their relationships to HIV management, among a diverse cohort.

**Methods:**

Participants (*N* = 271) received structured baseline assessments and HIV viral load testing. Primary outcomes were being well-engaged in HIV care and HIV viral suppression. A subset (*N* = 41) was purposively sampled for maximum variability for in-depth interviews. Quantitative data were analyzed with descriptive statistics and logistic regression, and used to develop a research question about life contexts. Qualitative data were analyzed with directed content analysis, and the joint display method was used to integrate results.

**Results:**

Participants were 25 years old, on average (SD = 2). The majority (59%) were Latine/Hispanic and the reminder African American/Black. Almost all were assigned male sex at birth (96%) and sexual minorities (93%). Half (49%) were born outside the US and 33% spoke primarily Spanish. They were diagnosed with HIV four years prior on average (SD = 3). Most were well-engaged in HIV care (72%) and evidenced viral suppression (81%). Speaking Spanish was associated with a higher odds of care engagement, and adverse childhood experiences and income from federal benefits were associated with a lower odds. None of the factors predicted viral suppression. Qualitative results highlighted both developmentally typical (insufficient financial resources, unstable housing) and atypical challenges (struggles with large bureaucracies, HIV disclosure, daily medication use). Federal benefits and the local HIV social services administration were critical to survival. Immigrant participants came to the US to escape persecution and receive HIV care, but HIV management was often disrupted. Overall qualitative results highlighted both risk and protective factors, and resilience. Qualitative results added detail, nuance, and richness to the quantitative findings.

**Conclusions:**

The present study advances what is known about the backgrounds and contexts of diverse and understudied AABL young/emerging adults living with HIV.

**Supplementary Information:**

The online version contains supplementary material available at 10.1186/s12889-025-21869-3.

## Introduction

The present study takes a mixed methods approach to describe a set of sociodemographic, background, and contextual factors, and their relationships to HIV management, among African American/Black and Latine (AABL) young and emerging adults living with HIV. While most studies with this population are conducted in clinical settings, this study focuses on those recruited from the community, and includes subgroups known to be understudied in research. Primary among these are individuals who do not evidence HIV viral suppression, along with other subgroups with barriers to the HIV care continuum and research engagement such as persons who recently migrated to the United States (US), those with serious socioeconomic deprivation, and individuals with high levels of medical distrust or fear of HIV status disclosure that might impede research participation [1–3].

Insufficient engagement along the HIV care continuum among younger people with HIV is a serious and persistent public health problem [4, 5]. The US public health leadership has set a goal of ending the HIV epidemic; that is, ending new HIV infections, by 2030 [6]. As part of this goal, 95% of people living with HIV must know their status, 95% of diagnosed individuals must be on HIV antiretroviral therapy, and 95% of those on HIV antiretroviral therapy must achieve HIV viral suppression [7]. These are called the 95-95-95 targets [7, 8]. Yet since the onset of the HIV epidemic, younger people living with HIV in the United States have consistently failed to meet these objectives. For example, among those ages 13–24 years diagnosed with HIV, 80% have received HIV care, 55% are retained in care, and only 65% are virally suppressed based on their most recent test [9]. Rates of engagement are similarly insufficient for the next highest age group tracked by the Centers for Disease Control and Prevention (CDC), those 25–34 years of age [9]. Rates of *sustained* HIV viral suppression are even lower. Sustained viral suppression is necessary for optimal health, longevity, a high quality of life, and to prevent transmission of HIV to others [10]. In a national study, among those living with HIV ages 13–29 years, only 42% sustained viral suppression [10]. Moreover, racial/ethnic disparities in engagement along the HIV care continuum and sustained viral suppression are marked. African American/Black persons in the 13-29-year age group have the lowest rates of sustained viral suppression (36%), followed by Hispanic/Latine persons (47%), compared to 51% of White persons [10]. But, the factors that promote or impede engagement along the HIV care continuum among younger people living with HIV are insufficiently understood, particularly among those with the lowest rates of engagement along the HIV care continuum, AABL persons [11].

The present study focuses on AABL persons living with HIV ages 19–28 years, the developmental periods from late adolescence/young adulthood through emerging adulthood [12, 13], with an emphasis on those who are understudied compared to their peers with fewer barriers to care and research [1–3]. In this paper we refer to this period as “emerging adulthood” for parsimony. AABL emerging adults living with HIV must grapple with numerous developmental tasks, meet milestones, and move towards autonomy, similar to their peers not living with HIV. These include typical milestones in domains such as education, work, and relationships [14, 15]. As AABL persons, they must also manage structural racism, stigma, and discrimination related to race/ethnicity, and for many in this population, sexual/gender minority status as well. Moreover, they have stressors and challenges not typical for this developmental period related to HIV; namely, adapting to an HIV diagnosis, and ongoing independent HIV management such as attending primary care appointments regularly and taking medication daily [16].

Social action theory guides the study. Social action theory is a comprehensive integrative framework to advance understanding of health-promoting behavior and health habits [17]. Social action theory expands the familiar social-cognitive frame by specifying how important goals and routines are shaped by the larger context. The theory proposes mechanisms by which environmental structures influence core goals for health, motivation, and problem-solving activities critical to self-change processes. These, in turn, ultimately drive action states such as limited occurrence health behaviors (namely, engagement in HIV care) and repeated or sustained protective health behaviors (HIV medication adherence leading to HIV viral suppression). For this program of research, we focused specifically on domains known to be or hypothesized to be salient for the populations of AABL young and emerging adults living with HIV. Thus, we refined the social action theory model for this population.

**Fig. 1:**
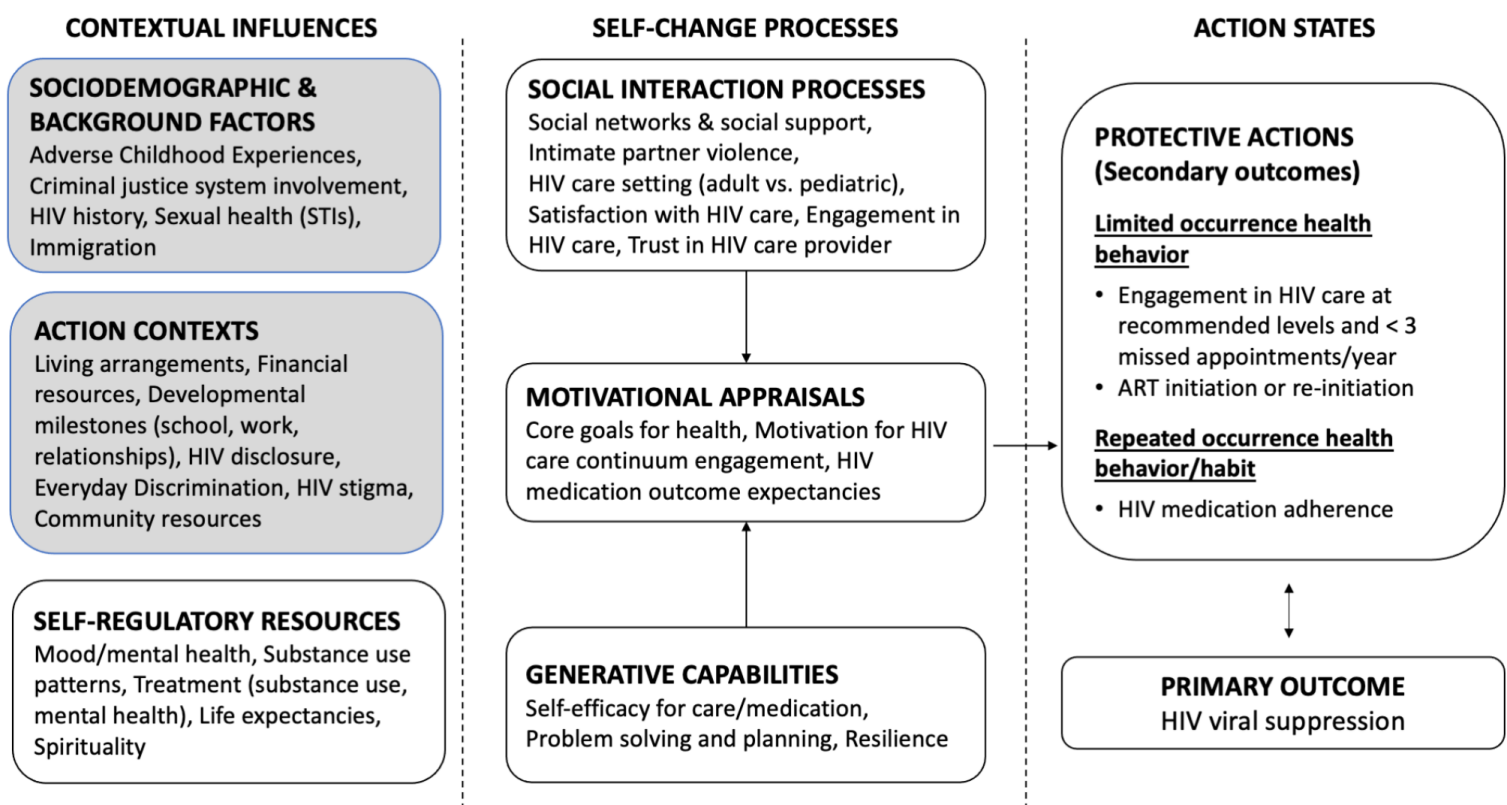
Social action theory model with the domains examined in the present study highlighted

The present study is descriptive and focuses on a subset of domains in our refined social action theory model, namely, contextual influences: sociodemographic characteristics, background factors, and action contexts. These contextual influences are important mainly “upstream” factors (both historical and current) that influence processes found “downstream” in the model. In the theory, self-change processes and behaviors (called action states) are shaped by a range of contextual influences related to socioeconomic status, the life history, and environmental stresses, supports, and resources. In the following sections, we highlight what past literature has found regarding the contextual influences explored in this study, including domains that have received relatively little attention in the literature to date among this population (e.g., adverse childhood experiences, everyday discrimination). The main domains explored in the present study are highlighted in Fig. 1, along with other domains in the model not explored here, for context (not all sociodemographic characteristics are included in the figure for parsimony).

Regarding sociodemographic characteristics, AABL persons are over-represented in the population of young and emerging adults living with HIV compared to their proportions in the general population, as are those with a non-heterosexual sexual orientation (i.e., gay, lesbian, bisexual, queer, etc.) and/or non-cisgender gender identity (i.e., transgender, gender fluid, gender non-conforming, gender non-binary, etc.) [11]. We describe in the present study a range of demographic characteristics, including a detailed assessment of gender identity that captures diverse experiences and terms.

Regarding background factors, we examine domains related to country of origin, immigration, and primary language (English or Spanish). Little is known about the immigration status of AABL young and emerging adults living with HIV, or about language barriers among immigrants, refuges, or asylum seekers, including those whose sole language is Spanish. Among adult persons living with HIV, Latine immigrants account for a third of all HIV diagnoses among Latines [18], Further, they are at greater risk than their US-born peers for delayed diagnosis and presentation to care [18, 19]. When in the US, Latine immigrants face numerous challenges that limit their access to healthcare services [18, 20, 21]. On the other hand, in the general population, a “healthy immigrant” effect has been observed, where immigrants in developed nations have better health outcomes than native-born citizens of the host country [22]. We also assess adverse childhood experiences (ACEs). ACEs are difficult and potentially traumatic events that occur in childhood before the age of 18 years, such as experiencing violence, abuse, or neglect. ACEs are well-known risk factors for a range of poor outcomes among adult persons living with HIV including poor mental health, quality of life [23], and substance use [24, 25]. However, their relationship to engagement along the HIV care continuum is less well-known for this age group. Last, involvement in the criminal justice system has been linked to poor HIV care and treatment outcomes among AABL young and emerging adults living with HIV [26–28], who are more likely to have been recently incarcerated than their HIV-negative peers [29, 30]. The present study will advance what is known about these background factors and HIV care continuum engagement in this age group.

Regarding action contexts, we focus on factors such as living arrangements, housing stability, work, and financial status. Lack of stable housing and insufficient economic resources have been linked to poor HIV care and treatment outcomes [26–28]. Young people living with HIV (aged 18–24 years) are more likely to reside in low-income households compared to their HIV-negative peers [29, 30]. We also assess a related set of domains to characterize developmental milestones in areas such as education, relationships, and work in the formal economy, as well as involvement in the street economy (e.g., sex work, drug dealing, burglary, panhandling). Little is known about street economy engagement in this population [1].

The present descriptive mixed methods study uses a sequential explanatory design [31]. This design proceeds in two phases where quantitative data are analyzed first, followed by qualitative data that explain and elaborate on the quantitative findings [31]. First, we explored sociodemographic, background (mainly historical), and action context domains using quantitative data. In particular, we described both those participants with HIV viral suppression and those without. Indeed, those who do not evidence HIV viral suppression are at serious risk for adverse health outcomes and poor quality of life, but are under-studied compared to their peers with viral suppression. Next, we investigated relationships between these domains and two outcomes: being well-engaged in HIV care and HIV viral suppression. Then, we used the quantitative findings to generate a research question that could be addressed with qualitative data, to advance understanding of the quantitative data. Last, we integrated the quantitative and qualitative data using the joint display method, and this integration informed the interpretation of the findings. These contextual influence are distal to the outcomes of interest compared to other factors in the model, and may interact with these other factors. Nonetheless, this paper aims to describe this under-studied population in detail and take the first step in exploring the complex factors that influence HIV management for this group.

## METHODS

The present study uses baseline quantitative and qualitative data from a longitudinal investigation of factors that promote or impede engagement along the HIV care continuum among AABL persons living with HIV aged 19–28 years, including those with and without HIV viral suppression. The larger study was conducted in New York City (NYC) and Newark, NJ. The study was conducted by an academic institution in NYC in partnership with a large multi-service community-based organization (CBO) in Newark, NJ called the North Jersey Community Research Initiative (NJCRI). NJCRI provides medical and social services for AABL young and emerging adults LWH. Between December 2021 and October 2023, we enrolled 271 individuals, 19% with non-suppressed HIV viral load. This constitutes nearly all (94%) of the 287 potential participants who completed the screening process and met all eligibility criteria. Participants engaged in a baseline assessment and HIV viral load testing at a commercial laboratory. A subset of participants engaged in semi-structured qualitative interviews. Activities were carried out in English and Spanish. Data were collected and stored using REDCap, a secure, web-based application designed to support data capture for research studies. In-person study activities took place mainly at a field site in lower Manhattan and also in Newark, NJ. (Study participants from NJ could easily travel to NYC or be seen in NJ.) The project’s field name was N4 Connect. Participants gave informed consent for study activities.

### Participants

Eligibility criteria were: age 16–28 years; AABL race/ethnicity; residence in the NYC or Newark, NJ metropolitan areas; HIV diagnosis (confirmed with medical documentation); diagnosed with HIV *≥* 3 months ago; HIV was transmitted behaviorally, not perinatally; and able to conduct activities in English or Spanish.

### Recruitment

We used a hybrid recruitment strategy informed by literature on recruiting populations that are hidden or are located in high-risk contexts and refined during our past studies. The hybrid sampling plan had the following elements: social media recruitment (both passive paid and free ads and active strategies using Snapchat, Facebook, Tik Tok, Instagram, and Reddit), classified advertisements (free newspaper called *amMetro*, Craig’s List), ads placed in public transportation venues (NYC subway), dating apps such as Grindr, Jack’d, and Positive Singles (both passive banner ads and active engagement on the app), peer-to-peer recruitment (participants provided their peers with coded coupons that linked the recruiter to the recruit, recruiters were provided with $15 compensation per referral), and recruitment in community-based organizations in NYC and Newark (both active and passive strategies using flyers, including referrals from providers). As we describe in more detail elsewhere, in addition to these multiple recruitment strategies, recruiting participants for the present study required time, repeated contacts, relationship building, creative engagement approaches, and consultation from professional market researchers [3].

### Procedures

#### Screening for eligibility

Screening took place in two stages. The first step was carried out by phone. Participants were contacted by study staff using information provided on the contact form or participants contacted the study by text or phone. Participants provided verbal informed consent and then were screened for eligibility. Screening was carried out using a brief structured assessment on REDCap (10 min). Locator information was obtained to facilitate future contact (phone number, email address, mailing address). Those meeting eligibility criteria proceeded to the next step which was carried out in-person at one of the study sites. Informed consent was obtained, HIV status was confirmed with medical documentation (using prescriptions, pill bottles, or a laboratory report) and HIV viral load level was assessed (to monitor the proportion enrolling in the study with suppressed versus non-suppressed HIV viral load). HIV viral load levels were assessed in two ways: participants could provide a laboratory report from their health care provider carried out in the past two months or could be escorted to a commercial laboratory and have a blood specimen drawn for HIV viral load testing. There was no compensation for the first screening interview and participants were compensated $25 for presenting for the second screening visit, and $40 if they provided the laboratory report or $25 for providing the blood specimen at the commercial laboratory. They were provided with funds for local round-trip transportation ($6).

#### Baseline assessment

Among those found eligible, signed informed consent was obtained for study activities. The baseline assessment included a structured assessment battery with Computer-Assisted Personal Interviews and Audio-Computer Assisted Self- Interview sections, taking approximately 60 min to complete. Participants were compensated $60 for the baseline assessment and received funds for local round-trip transportation.

#### Qualitative in-depth interview procedures

From the larger sample, participants were purposively sampled for maximum variability on key indices including language (English vs. Spanish), race/ethnicity, and HIV viral load status (suppressed vs. non-suppressed). A total of 41 interviews were conducted. The qualitative interviews lasted between 60 and 90 min and were conducted over the phone or in-person at a study field site. The interviews were carried out by four experienced Master’s and PhD-level qualitative researchers who were trained in public health or anthropology. Three qualitative researchers were fully bilingual in English and Spanish and 32% of the interviews (13/41) were carried out in Spanish. Interviews were conducted between six and twelve months post-enrollment, to allow for the capture of stability or change in HIV care continuum engagement patterns. A semi-structured template was used to guide the qualitative interviews. Interviews were audio-recorded and then professionally transcribed. Spanish transcriptions were translated into English by an automated (AI-based) professional service that was confidential and secure, and then transcripts were checked for accuracy by members of the research team who were fluent in Spanish. Participants were compensated $60 for the qualitative interview.

### Measures

#### Sociodemographic characteristics

We assessed age (in years), gender identity (e.g., man, woman, gender non-binary, transgender, gender fluid), sexual orientation (e.g., gay, lesbian, straight/heterosexual, pansexual, queer), race/ethnicity, and sex assigned at birth (male, female, intersex, other) [32, 33]. Data were recoded to indicate whether participants were cisgender or not (that is, gender identity corresponds with the sex assigned to them at birth; not transgender).

#### Background factors

We assessed country of birth (USA - mainland, Hawaii, Alaska; Puerto Rico; other North America, including Mexico; Central America; Caribbean; South America; Europe, including Eastern Russia; etc.), primary language (English or Spanish), immigration status (US citizen; permanent resident/green card; valid tourist visa/work visa or permit/student visa; refugee status, asylum, temporary protected immigrant status; undocumented, other), criminal justice system involvement (ever stopped by police, ever spent one or more nights in jail, yes/no), medical insurance, education (coded as less than high school vs. high school graduate or higher), and HIV history; namely, years since HIV diagnosis [32, 33].

Sexual health/sexually transmitted infections were assessed by self-report. We assessed whether participants had been diagnosed with the following sexually transmitted infections over the past year (no, yes, don’t know): chlamydia; genital warts, anal warts, human papillomavirus (HPV); gonorrhea; hepatitis B virus (HBV); hepatitis C virus (HCV); herpes, HSV1 or HSV2; syphilis; urethritis, or some other sexually transmitted infection [34].

We assessed the presence or absence of dimensions of childhood adversity prior to the age of 18 years using a revised version of the Adverse Childhood Experiences inventory called the ACES-R [35]. Using 14 items, the ACES-R extends the original 10-item ACEs measure and includes items on widely recognized childhood adversities (low socioeconomic status, peer victimization, peer isolation/rejection, and exposure to community violence), along with domains in the original ACEs measure: emotional abuse, physical abuse, sexual assault, emotional neglect, physical neglect, mother being treated violently, household substance abuse, household mental illness, parental separation or divorce, and incarcerated household member. The additional items have been shown to improve the prediction of physical and mental health outcomes [35]. Items are coded on a yes/no scale and the mean number of affirmative responses is calculated, which comprises the ACES-R score which ranges from 0 to 14. One participant skipped these items, and the missing ACES-R score was set to the sample median.

##### Action contexts

We assessed living arrangements (temporary [six months or less] or unknown stability or not) and financial resources (employment in the formal and street economy, income from welfare, public assistance, social security, disability, or workers’ compensation), the whether needs for necessities could not be met in past six months (rent, food, utilities) at least once [32, 33].

Developmental milestones were assessed including relationship status (e.g., single and not seeking a sexual or romantic partner or partners, single and seeking a sexual or romantic partner or partners, dating or “seeing” one or more persons, married), sexual behavior (ever sexually active), highest level of education, and work experiences (ever employed on–the-books, current employment status, sources of income, and engagement in the street economy) [32, 33].

HIV disclosure to friends or family was assessed using two items. The items were “How many of your immediate family members know your HIV status?” and “How many of your peers know your HIV status? Peers include your friends, co-workers, and schoolmates.” Items were assessed on a four-point Likert scale ranging from “1. none” to “4. all” and then re-coded to reflect whether at least some family *and* friends know the participant’s HIV status (yes/no).

Discrimination was assessed using the Everyday Discrimination Scale, which is comprised of nine items that assess experiences of discrimination such as being treated with less courtesy than other people are and people acting as if they are afraid of you. Items are assessed on a six-point Likert scale ranging from “1. never” to “6. almost every day.” Items were summed where higher scores indicate greater experiences of discrimination. The scale was reliable (Cronbach’s alpha = 0.90). The scale also asks the reasons for the discrimination: HIV status, race/ethnicity, sexual orientation, gender, age, height, national origins, etc [36]. Three participants skipped these items, and the missing total scores were set to the sample median.

HIV stigma was assessed with the HIV Stigma scale. This scale consists of 10 items that assess experiences such as “I feel that I am not as good a person as others because I have HIV” and “most people with HIV are rejected when others find out.” Items are assessed on a four-point Likert scale ranging from “1. strongly disagree” to “4. strongly agree.” Items were summed and higher scores indicate greater experiences of stigma. The scale was reliable (Cronbach’s alpha = 0.83) [37]. One participant skipped these items, and the missing total score was set to the sample median.

### Primary outcomes (HIV care continuum indices)

HIV viral load was assessed via laboratory report and coded on the log_10_ scale [38]. We present the mean and SD and also coded HIV viral load status as suppressed (< 200 copies/mL) or non-suppressed (> 200 copies/mL) [39].

We assessed engagement in HIV care. There is no gold standard to measure retention in care and the selection of a retention measure can be tailored to context [40, 41]. We calculated two indices: whether attended two or more HIV primary care appointments in the past year, an accepted minimum [42], and whether the participant missed three or more HIV primary care appointments past year without prior cancellation, a factor independently associated with mortality [43]. We used these data to create a variable capturing whether the participant was “well-engaged” in HIV care made up of attending at least two HIV care appointments and fewer than three appointments missed without canceling in advance in the past year (yes/no).

### Qualitative interview template

Qualitative interviews were guided by a semi-structured template developed by the research team, which included experts on AABL persons living with HIV, sexual and gender minorities, immigration, and the HIV care continuum. The interview guide was structured as a series of suggested questions and prompts. The main purposes of the guide were to understand the participant as a whole person; understand factors that promote or impede engagement along the HIV care continuum and their causes and meanings (What has stayed the same, what has changed, and why); and explore domains that receive little attention in the structured assessments (e.g., community resources, identity, structural and societal factors and influences and how they operate). The template directed the interviewer from general to more specific questions in sections. We describe relevant sections of the template here. The template started with general introductions (*Tell me a little about yourself*,* What’s one thing that we really need to know to understand you?*), then moved to understanding factors that promote or impede engagement along the HIV care continuum and their causes and meanings (*Since you joined the N4 Connect project*,* has anything changed with respect to HIV care? Medication? What do you think led to the change in your HIV medication taking? How often does your level of adherence change? What are the reasons it changes*?); factors that promote or impede HIV medication adherence patterns (*We want to understand WHY some people take HIV medication consistently [if and when they do that] and if they do*,* HOW they are able to do that [looking at all sorts of factors]. It can be hard to explain things like this sometimes*,* but we will ask you to try*.); and developmental challenges (*disclosure*,* relationships*,* sexual behavior*,* sexually transmitted infections*,* school/work*).

### Quantitative data analysis

We used descriptive statistics to summarize HIV viral suppression and care engagement outcomes, as well as socio-demographic and background characteristics, HIV and other health factors, and action contexts. We present data for the cohort as a whole, and for the HIV virally suppressed and non-suppressed subgroups. Because of the large number of variables, we did not screen variables at the bivariate level. We did simplify the logistic regression models described below by not including highly overlapping variables (e.g., immigration and Spanish as the primary language). We also calculated variance inflation factors, and the highest of those, 2.3 associated with Spanish activities, likely due to its association with race/ethnicity, did not suggest a problematic degree of collinearity.

To estimate associations between socio-demographic and background factors, HIV and other health factors, action contexts, and the two outcomes, we used binary logistic regression. For each outcome, variables were entered in two blocks, forming two models for comparison: (1) socio-demographic, background, and health history only; and (2) socio-demographic, background, health history, and action contexts. Organizing the variables into blocks allowed us to determine what action contexts added to background variables. Coefficients estimated by binary logistic regression are log odds ratios, and exponentiating the coefficients leads to odds ratios (ORs), which describe how a one-unit change in the explanatory variable multiplies the outcome variable odds. Associations were reported as ORs with 95% confidence intervals. Given the sample size (*n* = 271) and prevalence of HIV viral suppression, power was at least 80% to detect an odds ratio of 1.6 for continuous predictors and an odds ratio of 2.4 for unevenly distributed (e.g., 25% vs. 75%) categorical predictors. The R statistical computing program was used for the logistic regression analysis, including tests of significance and confidence intervals. All tests of statistical significance were two-tailed, and *p* < 0.05 was considered significant.

### Qualitative data analyses

Analyses of qualitative data followed a directed content analysis approach that was both inductive and theory-driven [44]. Analyses were carried out in the Dedoose platform. We started with an initial list of “start codes” and their operational definitions generated by the primary qualitative analyst. This initial start code list was informed by the theories and perspectives framing the study. Using this code list, a set of three Ph.D.-level qualitative analysts coded interview transcripts. During the coding process, codes were refined, clarified, and/or broadened. We resolved discrepancies in codes and coding between the data analysts by consensus. Then, the interview transcripts were recoded using the final coding frame. We formed an interpretive community to organize codes into themes and sub-themes in an iterative process. The interpretive community was led by the three primary analysts and included members of the research team and study investigators. The interpretive community included people who identify as cisgender men and women, people who are gender non-binary or gender-fluid, those fully bilingual in Spanish, and people from White, African American/Black, Asian, and Latine backgrounds [45, 46]. Methodological rigor of the analysis was monitored continually in several ways. An audit trail of process and analytic memos was maintained [47]. Analysts engaged in debriefing sessions approximately monthly with the interpretive community. The primary analysts and the interpretive community regularly attended to the potential effects of the team’s positionality related to power and privilege, sex, gender, race/ethnicity, citizenship status, health, and socioeconomic status throughout the data collection process through reflection and training that focused on how these factors might affect interviewing and data analytic processes [48, 49].

### Data integration procedures

We used the joint display method to integrate data, following procedures outlined by Fetters and colleagues [50]. A joint display is a visual tool that consist of a side-by-side visual presentation of results. The process of creating the joint display is intended to bring about new insights beyond the information gained from the separate quantitative and qualitative results. Thus, joint displays are both a method and a cognitive framework for data integration and facilitate the production of new inferences [50]. Data integration was carried out by the interpretive community. Beginning with the major quantitative findings, the interpretive community assessed areas of convergence and divergence between the quantitative results and the primary themes in the qualitative data analysis. To do so, we used an informational matrix to compare results at a granular level (finding-by-finding) [50]. The results from this data integration effort were summarized and presented in a joint display table.

## Results

### Quantitative results

Table 1 shows socio-demographic and background factors. We focus here on the sample as a whole, not the two subgroups. Participants ranged in age from 19 to 28 years (mean = 25 years; SD = 2 years). Most were Latine/Hispanic (59%). Almost all (96%) were assigned male sex at birth and the majority (66.1%) were cisgender (not transgender). Almost all (93%) identified as gay, lesbian, pansexual, bisexual or otherwise not heterosexual. Almost half (49%) were born outside the US or Puerto Rico, and a third (33%) engaged in activities in Spanish because Spanish was their primary or only language. The mean ACES-R score was 7 (SD = 4). A total of 80% had an ACES-R score of four or higher, an indication of significant childhood adversity [51]. Participants had been diagnosed with HIV for an average of 4 years (SD = 3 years). Almost all (99%) had taken ART in the past and the majority had health/medical insurance (91%). Approximately half (52%) had ever been stopped or harassed by the police and 41% had ever spent one or more nights in a jail, prison, or detention facility. The primary outcomes are also presented in Table 1. Almost all (92%) attended at least two HIV care appointments in the past year, and 21% missed three or more appointments without prior cancellation. A total of 72% were well-engaged in HIV care as we defined it, the primary HIV care outcome. A total of 19% of participants did not evidence HIV viral suppression at enrollment. HIV viral load on a log10 scale ranged from 2.30 to 6.13 copies/mL.

**Table 1:** Sociodemographic and background characteristics [mean (SD) or %, (N)]

	Overall (*N* = 271)	Suppressed (*N* = 219)	Not Suppressed (*N* = 52)
Current age, in years	25.2 (2.38)	25.2 (2.37)	25.1 (2.46)
Median [Q1, Q3]	25.0 [24.0, 27.0]	25.0 [24.0, 27.0]	25.5 [23.8, 27.0]
Range [Min Max]	[19.0, 28.0]	[19.0, 28.0]	[19.0, 28.0]
*Race/ethnicity*			
Latine or Hispanic	58.7 (159)	59.8 (131)	53.8 (28)
Black/African American or bi- or multi-racial (Non-Hispanic/Latine)	41.3 (112)	40.2 (88)	46.2 (24)
*Sex assigned at birth*			
Male	95.6 (259)	95.9 (210)	94.2 (49)
Female	3.3 (9)	2.7 (6)	5.8 (3)
Intersex/other/prefer not to answer	1.1 (3)	1.3 (3)	0 (0)
*Gender identity*			
Cisgender	66.1 (179)	64.8 (142)	71.2 (37)
Transgender, gender expansive, gender non-binary, gender queer or otherwise not cisgender	33.9 (92)	35.2 (77)	28.8 (15)
Lesbian, gay, bisexual, queer, or other non-heterosexual sexual orientation (LBGQ)	93.4 (253)	93.6 (205)	92.3 (48)
Born outside US or Puerto Rico	49.1 (133)	51.1 (112)	40.4 (21)
Engaged in activities in Spanish	32.5 (88)	35.6 (78)	19.2 (10)
ACES-R score (range 0–14)	7.19 (3.72)	7.28 (3.63)	6.77 (4.12)
Median [Min, Max]	8.00 [4.00, 10.0]	8.00 [4.00, 10.0]	8.00 [3.00, 10.0]
ACES-R scores 4 or more	79.6 (215)	82.1 (179)	69.2 (36)
Years since HIV diagnosis	3.90 (2.86)	3.81 (2.88)	4.27 (2.80)
Median [Min, Max]	3.00 [0, 14.0]	3.00 [0, 14.0]	4.00 [0, 12.0]
Range [Min, Max]	[0, 14.0]	[0, 14.0]	[0, 12.0]
Have taken HIV medication - lifetime	98.5 (267)	98.6 (216)	98.1 (51)
Has medical insurance	90.8 (246)	92.7 (203)	82.6 (43)
*Criminal justice involvement (lifetime)*			
Ever stopped or harassed by the police	51.7 (140)	47.9 (105)	67.3 (35)
Ever spent one or more nights in a jail, prison, or detention facility	40.6 (110)	37.9 (83)	51.9 (27)
*Outcome variables*			
Attended 2 or more HIV primary care appointments in the past year	91.5 (248)	95.0 (208)	76.9 (40)
Missed 3 or more HIV primary care appointments past year without prior cancellation	20.6 (55)	17.1 (37)	36.0 (18)
Well-engaged in HIV care in past year	72.3 (196)	77.6 (170)	50.0 (26)
Log10 HIV viral load at enrollment	2.63 (0.818)	2.30 (0)	4.01 (1.06)
Median [Q1, Q3]	2.30 [2.30, 2.30]	2.30 [2.30, 2.30]	3.94 [3.03, 5.04]
Range [min, max]	[2.30, 6.13]	[2.30, 2.30]	[2.39, 6.13]

Table 2 shows action contexts and we present select results here. Half (51%) were in a living situation considered temporary. The most common relationship status was being single, but not seeking a sexual or romantic partner or partners (37%). Most (82%) had a high school degree or higher and the majority (71%) had worked in the formal economy at some point in their lives. Approximately half (57%) were receiving income from welfare, public assistance, social security, disability, or workers’ compensation. Approximately half (55%) had worked in the street economy in their lives (e.g., trading sex for money, drugs, food, or a place to stay, stealing or shoplifting something from a store, or dealing, selling, bagging, or running drugs) and 43% had done so in the past six months. Most (82%) experienced financial hardship where they could not meet needs for necessities (rent, food, utilities) in past six months. Disclosure of HIV status was moderate, where approximately half (53%) disclosed to at least some family and friends. The mean score on the Everyday Discrimination Scale was 25 (SD = 13, range 9–54). The most common reasons for discrimination were sexual orientation, gender, HIV status, and race. The mean score on the HIV stigma scale was 26 (SD = 7, range 10–40). The odds of viral suppression were about 3.5 times higher among participants who reported being well-engaged in HIV care, as defined above (Fisher’s Exact Test OR = 3.45, 95% CI 1.75–6.82, *p* < 0.0001, data not on Table 2). For parsimony, we provide more detail on these sociodemographic and background factors and action contexts in Supplemental Tables 1–3.

**Table 2:** Action contexts

	Overall (*N* = 271)	Suppressed (*N* = 219)	Not Suppressed (*N* = 52)
Living arrangement is temporary (6 months or less) or unknown stability	51.3 (139)	53.0 (116)	44.2 (23)
Most common relationship status: Single, not seeking a sexual or romantic partner or partners	36.5 (99)	36.5 (80)	36.5 (19)
High school graduate/GED or higher	82.3 (223)	84.5 (185)	73.1 (38)
Ever been employed on-the-books (a job where you are paid with a check)	71.2 (193)	69.9 (153)	76.9 (40)
Current employment status is not working but actively looking	52.8 (143)	51.1 (112)	59.6 (31)
Has worked in the informal economy in past 6 months	43.4 (66)	42.4 (50)	47.1 (16)
Income from welfare, public assistance, social security, disability, or workers compensation	57.2 (155)	57.1 (125)	57.7 (30)
Ever engaged in the street economy– lifetime	55.4 (150)	53.9 (118)	61.5 (32)
*Financial hardship*			
Could not meet needs for necessities in past 6 months (rent, food, utilities), at least once	81.9 (222)	81.3 (178)	84.6 (44)
*HIV disclosure*			
At least some family AND friends know HIV status	52.8 (143)	52.5 (115)	53.8 (28)
Everyday Discrimination Scale score (range 9–54)	24.5 (11.1)	24.2 (10.6)	25.4 (13.0)
Median [Min, Max]	22.0 [9.00, 54.0]	23.0 [9.00, 54.0]	21.5 [9.00, 54.0]
*Main reason for these experiences*			
Your sexual orientation	60.1 (163)	58.9 (129)	65.4 (34)
Your gender	45.4 (123)	44.7 (98)	48.1 (25)
Your HIV status	37.3 (101)	37.0 (81)	38.5 (20)
Your race	34.3 (93)	32.0 (70)	44.2 (23)
Some other aspect of your physical appearance	31.0 (84)	30.6 (67)	32.7 (17)
Your shade of skin color	26.9 (73)	25.6 (56)	32.7 (17)
Your ancestry or national origins	22.5 (61)	23.7 (52)	17.3 (9)
Your age	21.0 (57)	22.4 (49)	15.4 (8)
Your education or income level	18.8 (51)	17.4 (38)	25.0 (13)
Your weight	17.7 (48)	17.4 (38)	19.2 (10)
Your height	12.9 (35)	12.8 (28)	13.5 (7)
HIV stigma (range 10–40)	25.8 (6.51)	25.7 (6.62)	26.3 (6.09)
Median [Min, Max]	26.0 [10.0, 40.0]	26.0 [10.0, 40.0]	26.0 [12.0, 37.0]

Table 3 shows the results of multivariable logistic regression with being well-engaged in HIV care as the outcome. In the model with background factors only, completing study activities in Spanish was associated with increased odds of care engagement (OR = 2.7, *p* = 0.02) while adverse childhood experiences were associated with decreased odds of care engagement (OR = 0.91, *p* = 0.021). In the model with action context variables added, receiving income from government sources was associated with decreased odds of HIV care engagement (OR = 0.50, *p* = 0.038).

**Table 3:** Multivariable logistic regression models for the HIV care outcome (well-engaged in HIV care)

	Model 1	Model 2
Age at enrollment	0.949 [0.827, 1.086]	0.959 [0.829, 1.105]
	0.454	0.567
Years living with HIV	0.937 [0.842, 1.043]	0.965 [0.860, 1.084]
	0.233	0.548
Cisgender	0.678 [0.354, 1.272]	0.616 [0.299, 1.236]
	0.233	0.179
LGB sexual orientation (non-heterosexual)	1.465 [0.800, 2.689]	1.416 [0.736, 2.728]
	0.216	0.297
Black or multiracial race/ethnicity (non-Hispanic)	0.885 [0.449, 1.725]	0.773 [0.381, 1.547]
	0.721	0.470
**Engaged in activities in Spanish**	**2.660 [1.182**, 6.186] *	1.872 [0.674, 5.416]
	**0.020**	0.235
Education: Less than high school	0.929 [0.446, 2.006]	1.029 [0.458, 2.397]
	0.848	0.945
Not enough money in the past six months for rent, food, or utilities	0.526 [0.218, 1.171]	0.632 [0.254, 1.459]
	0.131	0.299
**Number of Yes responses to ACES items (0–14)**	**0.909 [0.837**, 0.984] *	0.956 [0.871, 1.046]
	**0.021**	0.329
Housing arrangement temporary or of unknown stability		1.690 [0.923, 3.130] +
		0.091
Disclosure: At least some family AND friends know HIV status		0.860 [0.463, 1.589]
		0.630
Ever stopped by police		0.902 [0.461, 1.756]
		0.760
Ever spent one or more nights in jail		1.190 [0.618, 2.330]
		0.606
Everyday Discrimination Total Score (9–54)		0.979 [0.949, 1.009]
		0.170
HIV Stigma Total Score (10–40)		0.963 [0.914, 1.014]
		0.155
Ever been employed on-the-books		1.334 [0.562, 3.178]
		0.512
Worked in the street economy - lifetime		0.736 [0.369, 1.451]
		0.377
**Income from welfare**,** public assistance**,** social security**,** disability**,** or workers compensation**		**0.497 [0.254**, 0.954] * **0.038**
Single, not seeking a sexual or romantic partner or partners		1.258 [0.673, 2.393]
		0.476

Estimates are odds ratios with 95% confidence interval; a *p*-value appears below each interval estimate

Table 4 shows the results of multivariable logistic regression models with HIV viral suppression as the outcome. In both models, none of the variables reached a conventional level of statistical significance.

**Table 4:** Multivariable logistic regression models for HIV viral load suppression

	Model 1	Model 2
Age at enrollment	1.014 [0.871, 1.176]	1.017 [0.870, 1.186]
	0.857	0.831
Years living with HIV	0.955 [0.846, 1.077]	0.957 [0.842, 1.087]
	0.447	0.493
Cisgender	0.659 [0.313, 1.333]	0.705 [0.318, 1.509]
	0.256	0.376
LGB sexual orientation (non-heterosexual)	1.643 [0.842, 3.223]	1.409 [0.690, 2.882]
	0.145	0.344
Black or multiracial race/ethnicity (Non-Hispanic)	1.197 [0.570, 2.487]	1.163 [0.548, 2.442]
	0.630	0.691
Engaged in activities in Spanish	2.340 [0.969, 5.900] +	1.776 [0.580, 5.757]
	0.063	0.324
Education: Less than high school	0.513 [0.244, 1.112] +	0.456 [0.200, 1.057] +
	0.083	0.063
Not enough money in the past six months for rent, food, or utilities	0.711 [0.280, 1.642]	0.707 [0.273, 1.673]
	0.446	0.449
Number of Yes responses to ACES items (0–14)	1.043 [0.957, 1.137]	1.058 [0.959, 1.166]
	0.338	0.259
Housing arrangement temporary or of unknown stability		1.263 [0.651, 2.469]
		0.491
Disclosure: At least some family AND friends know HIV status		1.228 [0.633, 2.390]
		0.544
Ever stopped by police		0.535 [0.243, 1.136]
		0.110
Ever spent one or more nights in jail		0.753 [0.368, 1.541]
		0.435
Everyday Discrimination Total Score (9–54)		1.006 [0.971, 1.043]
		0.751
HIV Stigma Total Score (10–40)		0.992 [0.938, 1.049]
		0.790
Have you ever been employed on-the-books		1.042 [0.392, 2.694]
		0.933
Involved in street economy - lifetime		0.813 [0.385, 1.693]
		0.583
Income from welfare, public assistance, social security, disability, or workers compensation		1.247 [0.614, 2.536]
		0.540
Single, not seeking a sexual or romantic partner or partners		0.797 [0.401, 1.602]
		0.519

Estimates are odds ratios with 95% confidence interval; a *p*-value appears below each interval estimate

### Developing the qualitative research questions

The research team used the quantitative findings to generate a set of research questions that could explain and extend the quantitative findings and that could be reasonably addressed using the qualitative data. We also attended to domains that were not included in the quantitative data set but that aligned with the conceptual model (e.g., community resources). Further, we sought to focus on domains that are understudied in the literature or that we thought could advance the literature. We considered results that met or approached statistical significance at a *p* < 0.10 level. Quantitative results highlighted a number of challenging life contexts in domains such as housing, work in the formal and informal economies, and financial hardship, and protective factors such as government benefits (although these were negatively associated with being well-engaged in HIV care). Further, immigration experiences were common. The majority of participants were taking HIV medication to the point of HIV viral suppression, suggesting supportive contexts and resilience. The team determined this warranted exploration. Thus, the research questions we developed were focused on life contexts, both past and present. The specific questions were: How do various life contexts that quantitative results suggest are important, such as housing, work, financial resources, and immigration experiences, operate and how do they support or impede engagement along the HIV care continuum (both engagement in HIV care and HIV medication adherence)? What are participants’ perspectives on community resources (e.g., involvement in various community-based organizations), which are not included in the quantitative model but may be important, and their possible benefits? What are participants’ views on adverse childhood experiences and their effects? Consistent with a mixed methods approach, this qualitative analysis was not intended to be broad or comprehensive, but instead was focused on a discrete set of domains and questions in order to add depth to, and explain and enhance, the quantitative findings.

### Qualitative results

#### Overview

Participants described the features of a number of important life contexts, including how these contextual factors promoted or impeded wellbeing and engagement along the HIV care continuum. Participants had relatively little to say about engagement in HIV care, but HIV medication adherence was an important and ever-present factor in their lives, whether they were currently taking HIV medication or not. Results were interpreted in light of participants’ age and developmental levels. From a developmental perspective, it is not typical for young and emerging adults to be grappling with a chronic health condition, particularly a complex and stigmatized condition, or to be engaging in medical care regularly and taking medication daily, while also interfacing with the large bureaucracies that administrate benefits for persons with limited income and/or living with HIV. In addition to these HIV-specific tasks, participants were managing the types of challenges that persons their age generally contend with, such as work, finances, and housing *(“all the trials and tribulations”*). We found it was not common for participants to be enrolled in school or to discuss education. All or almost all participants in the present study had serious financial difficulties, which shaped contextual factors such as housing stability. Yet in the context of this confluence of developmentally typical and atypical challenges, it is notable that participants were by and large successful, at the time of the interview at least, in overcoming obstacles, thereby finding housing, meeting financial needs (even if just barely), and taking HIV medication with high levels of adherence with minimal disruptions. They did, however, reflect on recent past periods when wellbeing and HIV management were not so favorable, and these data yielded rich insights.

In the sections below we present participants’ perspectives on life contexts and how they might affect engagement along the HIV care continuum. As a reminder, nearly half of participants were born outside the United States, and a substantial proportion of these were refugees, asylum seekers, had temporary protected immigrant status, or were otherwise undocumented (data in Supplementary Table 1). A substantial proportion spoke Spanish as their primary or only language. This results section is organized around the following themes: housing, employment and finances, government benefits and the local HIV services administration, connections to community resources including lesbian, gay, bisexual, transgender, and queer (LGBTQ) resources, and immigration as a contextual factor. In the transcripts, participants did not generally volunteer or describe experiences that reflect the specific ACEs domains. However, experiences of discrimination and trauma were common, generally more recently than in childhood, although the type of trauma was not generally specified and only alluded to. For these reasons, we do not have a separate section on ACEs below. Names below are pseudonyms and identifying details (age, race/ethnicity, gender) are not included to protect confidentiality. We use the gender-neutral pronoun series they/them/theirs to describe participants.

#### Housing

Housing was one of the greatest challenges that participants faced, and was fundamental to wellbeing and HIV management. Some participants lived with their family of origin, but it was common for participants to elect not to live with family, for example because of the desire to live independently. Others were unable to live with family, such as in cases of tension, estrangement (“we had a bad fallout”), and/or family economic difficulties. In some cases, participants were distant from families, in part to avoid disclosure of their HIV status and/or sexual orientation. Participants could obtain housing on the open market, or receive housing assistance through the local HIV social services administration (namely, placement in supportive housing for people living with HIV such as single-room occupancy [SRO] residences, scattered site housing, or rent support) [52]. Emergency shelters, both HIV specific and general, were another housing option. Whether receiving HIV social services support or not, participants reported that unstable or inadequate housing was common, and this proved to be a significant barrier to wellbeing. In turn, diminished wellbeing and instability both could challenge, complicate, or interfere with HIV medication adherence. In particular, poor-quality housing (e.g., a lack of privacy) or having to move frequently commonly precluded participants from prioritizing HIV medication, at least for periods of time. In these cases, participants had to work out how and when to transport and take their HIV medications. This could require complex decision-making, forethought, and planning (e.g., weighing the risks of disclosure of one’s HIV status vs. the costs of missing a dose). When asked whether there had been times where it had been hard to take their medications consistently, Hugo described about a period of housing instability and its effects on HIV management:

*I was living with friends from time to time. Different friends. […] Because I was not home with my mother after we had like a bad*,* you know*,* fallout. Yeah. It’s been pretty tough. […] Before I got my apartment […] that was kind of hard because it’s like I kept leaving it [medications] at my mom’s house because I didn’t want to take it with me*,* because I didn’t know if I would lose it. Or*,* you know*,* people sell it. […] I’m staying at people’s houses and I’m sleeping over and waiting to see when I get my apartment. [.] I try to keep it [medication] in a place like where I know I’m going to remember or like sometimes in my bag I would put one extra just in case. Then I realized that’s kind of not a good way to keep it either*,* because sometimes I’ll lose it or [it will] just fly out. And maybe I didn’t want somebody to know what that was. […] That was kind of tough*,* you know*,* to manage at that time. But besides that [time]*,* I would say daily*,* I take it [HIV medication]. But sometimes I might miss like one or two [doses]*,* you know*,* like if I go out or something or I just forget.*

Thus, Hugo highlighted how difficult it can be to maintain high levels of HIV adherence in the absence of a private and stable living arrangement. Their quote further highlights the need for planning and forethought when needing to take medication daily while moving to different living circumstances, as well as concerns about disclosure of one’s HIV status, and the fear that HIV medications would be stolen and sold. Conversely, medication adherence was much less challenging when housing was private and stable, but never easy (“*I just forget”).*

Thus, housing circumstances could create obstacles to wellbeing and HIV management and cause stress. For example, housing could be very far from HIV clinics, neighborhoods could be experienced as unfamiliar and not particularly welcoming to younger people, and some had negative interactions with supportive housing staff. Nonetheless, it was common for participants to describe maintaining high levels of HIV medication adherence even in these types of less-than-optimal housing contexts. Dante described continuing to take HIV medication, even through periods of homelessness and loss of a family member:

*I was going through homelessness at the time*,* and I was in the shelter. And then also my grandmother passed away. So*,* this was a lot of things. And so*,* I gave everything a time for me to heal and get back to doing the things that were important to me and things that make me genuinely happy*,* if that makes sense.*

*Interviewer: Do you feel like that affected your engagement in HIV care at all?*

*Actually*,* it did not. It didn’t affect that. So a lot of times I’m pretty like*,* strong when it comes to things. And I always like to still take care of myself in the process. I have friends or people that I know and associate with that has not been as strong as I have when it comes to certain situations. And I feel like that’s something that I learned growing up from my grandmother because she was a very*,* very strong person and she barely even showed emotion to what was going on*,* even though I felt something in my gut. But she always handled it in a loving and surviving type of way. So I kind of keep that with me. And how do things*,* you know*,* I don’t cope with anything. I just cope with life.*

Although Dante was able to maintain medication adherence through periods of hardship, it was also common for participants to be unable to prioritize health and mediation during these types of transitions.

Participants generally preferred individual or independent housing to SROs, where privacy was limited and kitchens and bathrooms were shared. But, SROs were often a critical first step in obtaining a future optimal housing situation, and were certainly preferable to being unhoused. Typically, housing circumstances were linked to general wellbeing. Terrance described the importance of stable housing on wellbeing, and the critical role of the SRO, despite its imperfections:

*Mentally*,* I’m at a place in my mind where I’m at peace. Yes. That’s almost when I say I seem calm or stress free. It’s because I’m stressed when I don’t have the stability. So*,* if I’m stable or have a stable place*,* I don’t have to worry about where I’m gonna lay my head every night or where my next meal is gonna come. I’m actually good. […] [It was not always the case.] […] Before I got my SRO. Then I got an SRO [and] it was moreso trying to get a place of my own. But once that happened*,* I was*,* yeah. Everything seemed to work out from there. […] [As far as the SRO] I maybe only went there to sleep*,* but I guess it was OK. I’m very private*,* I like my personal space. […] Because we shared a bathroom [in the SRO]. I think the only thing that was*,* the only issue I had really mainly was the checking in [with SRO staff]. And again at one point*,* all the bathrooms were like*,* broke.*

Some participants were placed by the HIV social services agency in residential settings specifically for persons living with HIV (e.g., shelters or SROs specifically for individuals diagnosed with HIV). This type of housing placement was generally described as supportive and as reducing stigma, despite the risk for stigma from the external community as a result of being in a location exclusively for people with HIV. For some, these types of settings for persons living with HIV promoted comfort around taking HIV medication, since residents did not have to be concerned about inadvertent disclosure of their HIV status or fear experiencing stigma from others because of their HIV status. For example, Angelina described how they felt comfortable being open about their HIV status and taking HIV medication in the shelter in which they were residing because it was a housing setting expressly for persons living with HIV.

*I’m in a shelter where everyone is [HIV] positive*,* so I don’t [worry about other residents seeing my medication]. So*,* I’m like*,* they’re right here. Like*,* “Hello!”*,* because everybody knows you have to actually be positive to be here. So*,* like*,* that’s what makes me more comfortable as well. So*,* it’s like I’m not ashamed*,* you know. Like*,* “Hey*,* we’re taking them and we’re undetectable”.*

Overall, it was challenging for participants to manage their housing placements, in part because of financial precarity (described below) and difficulties navigating complex bureaucracies, and also likely reflecting the high cost of housing in the urban areas where the study took place. Residing in a stable and high-quality housing placement had a major positive effect on wellbeing, and at the same time, unstable or suboptimal placements took an emotional toll. Through social networks, family, and the HIV social services administration, participants were able to locate housing, even if not permanent or ideal. Indeed, the HIV social services administration played a vital role in housing placements and many participants would have been unhoused without those services and support. Notably, stable housing certainly contributed to HIV medication adherence from participants’ perspectives, and fostered routines (“*Because I get up*,* I go brush my teeth*,* I wash my face*,* I take my meds and then I take my shower. So it’s like a part of the routine.*”) Yet some participants described maintaining medication adherence patterns even when their housing circumstances were not optimal, highlighting, data suggest, durable intrinsic motivation for health, strong adherence habits, and resilience, along with faith and spiritual beliefs *(“I thank God for that every day*”).

#### Employment and finances

Participants described meeting financial needs through a combination of work in the formal (on-the-books) and informal (off-the-books) economies, “side hustles,” government benefits, and by relying on family and friends. Those with serious financial limitations and/or those unable to work in the formal economy (e.g., because of a lack of documentation) were often involved in the street economy to some extent. Further, participants could be involved in all of these various economies as they “hustled” to survive. Those who were undocumented could work “under another person’s papers,” but that was not considered ideal. It was not uncommon for participants to sell their HIV medication to corrupt pharmacies, a type of street economic activity. (This is illegal and puts pharmacies at risk for legal action.) In the quantitative data, we found over half of participants described themselves as not working in the formal economy but actively looking for work, and a substantial proportion worked in the informal and/or street economies. While some participants were working in satisfactory employment settings, generally work situations were precarious *(“I just lost my job. Like it hasn’t been the best situation*,* but*,* you know*,* life”*). Finding and maintaining work was challenging for participants, which is not uncommon for persons in this age group [53]. However, work was a vital source of stability, provided funds for housing and other essentials, and supported wellbeing and routines, including HIV medication routines, similar to the effects of high-quality stable housing. It was noteworthy that participants did not focus much, if at all, on being a person living with HIV when reflecting on their employment status and aspirations. Some participants were optimistic about their job prospects (“*So I took a little break from work*,* and now I’m ready to go back to work. And I’ve been looking for jobs. Yeah*,* it’s exciting”).* Rafael, who spoke Spanish as their primary language, described the combination of sources of income that allowed them to make ends meet, in the context of an ongoing asylum case.

*The government also gives us support every 15 days. Honestly*,* sometimes we have [run out of funds and] there is a friend who has*,* for example*,* a barbershop and sometimes we go*,* we sweep*,* we clean*,* or we do little jobs that he needs and he gives us something to eat*,* he gives us a little money [at] times. […] We have not been able to get a lawyer [to resolve immigration issues] or he doesn’t call us back. […] It makes us desperate*,* yes*,* it’s something that makes us desperate. […] But well*,* calmly we will continue in the fight.*

Again, social safety net services and resources were critical for participants’ survival, albeit insufficient or inadequate at times.

For the most part, participants did not experience discrimination in the workplace related to their HIV status. Those with a transgender or gender expansive gender identity did experience challenges finding work in the formal economy. For example, Carmen reported being pushed toward involvement in sex work because they were routinely denied jobs in the formal economy due to, they believed, their transgender gender identity.

*Like*,* day to day is*,* like*,* almost easing into sex worker life*,* because I never had to do that ever in my life. But that’s how hard it is to find a job and to live my life comfortably with the means to without being*,* you know*,* shunned in every spot that it takes to gain income besides anywhere [they want me to take] my wig off. […] That’s why I say [I’m[ struggling*,* because I know for a fact it’s like*,* not really easy. I don’t even know how to sex work*,* to be honest. I can’t even make money like the way that they say. I’m not even*,* I’m not experienced in it. My dad would kill me if he found it out.*

Overall, economic survival required a combination of activities in the formal, informal, and sometimes street economy, along with government benefits. Participants tended to lose jobs with some frequency and then needed to find new opportunities. Participants did not generally see living with HIV as barrier to employment or experience discrimination based on their HIV status in the workplace. However, factors such as a transgender gender identity and lack of documentation after immigration certainly did impede employment. Those who lost jobs commonly missed HIV medication doses or took a lengthy break from medication due to disruptions in life routines.

#### Government benefits and the local HIV social services administration

As noted above, because they were diagnosed with HIV and had financial need, participants were eligible for income support, housing, and other government benefits through the local HIV social services administration. These services and resources were seen as vital. But, this large and complex system was, not surprisingly, challenging for them to navigate. It was common for participants to have moved to the study’s geographical location from other domestic or international settings, sometimes in the hopes of receiving better HIV care. Commonly, benefits, including medical insurance, were disrupted during the time of the move or it was necessary to apply for benefits and access new health care settings upon arriving in the study’s geographical location. However, there were inconveniences and long waits for appointments with health care providers, as Terrance described:

*The only time I took one [a break from HIV medication]*,* I think I just took it [a break] twice. Once when my mother passed and when […] they wanted to change up my meds ‘cause they felt like they weren’t working anymore. But that was more so dealing with insurance and having to get it reinstated. And then*,* yeah*,* and moving up here. When I moved up here*,* it was a lot ‘cause I had to get my labs transferred*,* which took forever. So*,* I just started all over. […] So*,* I had to wait to get into health care*,* and get all appointment with the doctor.*

But regardless of whether participants had moved to the geographical region where the study was located or grown up in the area, they described how applications for services were commonly delayed or denied, sometimes insurance could be cancelled, and relationships with medical clinics could be challenging to manage. It was generally not clear to participants why claims were delayed or denied. Jalen described their path to consistent engagement along the HIV care continuum as having been difficult. Although they were currently successfully managing their benefits and insurance, they had spent a great deal of time getting information *(“I’ve went through needless hours of research on my own and several phone calls and digging up myself to get answers and to get help for myself because I was told no”*) and, for example, had experienced unexpected hassles keeping prescriptions filled:

*You know*,* my life is in your [health care/insurance system’s] hands. […] But again*,* day to day*,* it’s still a battle. Rather it’s […] with the medical providers or insurance not covering [medications] and saying that you have overused your medicine. You get it at the same time every month*,* you know*,* it doesn’t make sense [to say you overused].*

Those who had immigrated to the US had additional challenges obtaining benefits and services, particularly in cases where documentation and paperwork were lacking. In the experience of Andres, who spoke Spanish as their primary language:

*I came here in February*,* February 1*,* and I was already here in New York. I was here*,* I came here to the shelter*,* they did a lot of things to me*,* I mean*,* I slept on the street here too because I didn’t have the money. I had run out. Because I couldn’t find a way to be in a shelter*,* I’m still in the shelter. I’m fighting for [the HIV social services administration’s] support*,* unfortunately [the HIV social services administration] has denied me support for two months.*

Although the HIV social services administration was certainly a large and complex bureaucracy that participants considered challenging to navigate, it was common for participants to appreciate the services provided and compare services in the study’s geographical area favorably to other locations. As Pablo, who spoke Spanish as their primary language, described:

*I wanted to ask for a shelter in California and they also told me that there was a waiting list*,* and well*,* all those types of support are what make New York different here because I arrived here in New York and the next day I was already with medication*,* I was already with shelter*,* I was already with food support […] I no longer felt so alone because well*,* imagine [being] alone in such a big city and not having no type of support and for them to come here and provide that type of*,* that type of support makes you comfortable because it doesn’t make you feel so so bad.*

However, while essential for survival, benefits were considered insufficient to meet needs, as noted above. Jordan described these struggles, including how they managed to meet food needs by coordinating with a friend:

*I’m currently not working. I do receive food stamps*,* $250*,* and I get $180 in cash [each month]. That it’s not really working [not enough] but because of my friend letting me stay with her*,* it’s been working out pretty well. When I get my $250 and $180*,* she gets her food stamps literally*,* like a week or two weeks after mine. So we’ll have food in here for at least two and a half weeks. For at least two weeks. And then next week she’ll be able to get food again and put more food in.*

Overall, the extent to which participants highlighted their dependence on government benefits and social services cannot be over-stated. These resources were critical to their survival, wellbeing, and ability to engage along the HIV care continuum. But, resources were generally seen as insufficient to meet need and the benefits system, including the HIV social services administration, both large bureaucracies, were challenging to navigate.

#### Connections to community resources

As noted above, participants faced a great number of challenges, including challenges that persons their age do not typically grapple with. Yet as sexual and/or gender minority individuals, AABL persons, persons living with HIV, and in some cases, new immigrants, participants had the potential to access a variety of supportive community resources and CBOs. In fact, participants found connections to religious communities, the house and ball community (an underground queer subculture founded by Black transgender and queer persons, in which people “walk” [i.e., compete], perform, dance, lip-sync, and model), the network of CBOs for young people at-risk in the area, and networks of like-minded people. The formal and informal LGBTQ community was described as the largest and most well-organized of the communities. Thus, we explored participants’ perspectives on resources found from and within the LGBTQ community.

Participants were split regarding connections to these LGBTQ community resources. It was common for participants to not feel connected to or engage with the LGBTQ community. The reasons for this included not feeling accepted and feeling judged, and that the community was “pushy,” “catty,” “competitive,” and “toxic,” in large measure related to one’s appearance (*“And there’s always like*,* you know*,* some issue with how people look. And just like a judgment. But just constant*,* you know?”).* Caleb noted that although they had been involved in the LGBTQ community in the past, this was no longer the case:

*Sadly to say*,* it’s a very toxic community because it’s like everyone’s like trying to be whatever or in a competition of I don’t know what. […] And that’s stuff that I do not need in my life because I got way too many other issues*,* bills and things to take care of than to be worried about how*,* you know. Yeah. Mhm. So yeah*,* I don’t really associate myself much with the LGBTQ community anymore as much as I used to. If I see them*,* I say “Hi. Bye. How you doing? You’re doing all right? That’s good.” […] Sometimes I think a lot of people are also stuck up in their ways. So*,* people don’t even evolve. They just get stuck in whatever sometimes time*,* what do you call it? Time? Like century? Like not a century. A decade. Everybody has their own little decades. So some people get stuck in their decade and yeah. So*,* I evolve.*

In some cases, participants did not see themselves as defined by labels or identities, and therefore did not particularly or exclusively wish to engage with the LGBTQ community, as Ethan described:

*I identify as human*,* though that may be cliché. I don’t place myself in any specific group. […] And then as far as communities*,* like yes*,* I know I’m into dudes. But I like females sometimes. So I don’t say oh I’m gay or I’m straight or I’m bi. I’m just*,* like*,* they ask you what you are*,* I’m human. […] I feel very much supported by every community. I feel a part of the straight community and the gay community. So I just– I feel openly supported and accepted by all.*

On the other hand, many other participants were highly engaged in the LGBTQ community and used community resources. Regarding those who felt materially and emotionally supported by the LGBTQ community, some described attended large events and having positive experiences in the house and ball world. Some reported learning more about the importance of HIV medication adherence and HIV care through chosen family and other older members of the LGBTQ community. Dante described how a community member provided valuable guidance on the importance of HIV medication:

*I have like a trans mom in the community*,* and she always shared her experience about what happened to her that she was living her life and like*,* “Oh*,* I’m not going to take my medication and no*,* no*,* no.” And*,* you know*,* she did it for a very long*,* long period without taking it. And she didn’t think that nothing was happening because to her she felt good*,* you know*,* and she shared her story with us because she has kids. She considers us her children and it just was frightening and scary when she told us she was hospitalized because it had gotten so bad from not taking them for a very*,* very long period of time. And when she told us*,* told me*,* that story*,* it just made me think about it. And then I heard stories from people that live at the shelter that went through it. People that I cared about*,* that went through it*,* and it was just so scary. You know*,* it makes me learn from that.*Still other participants reported feeling empowered to serve as a role model for other LGBTQ younger people who might not feel comfortable being open about their sexual orientation and/or gender identity. Malik noted:

*I mean*,* just I feel like just being Black and gay and making sure*,* like*,* I just kind of want to be representation that I didn’t have. So yeah*,* just like*,* really try to amplify that part of my identity because I didn’t really see anybody doing that when I was younger*,* you know*,* and there are a ton more people doing it now. But still*,* I want the younger version of myself or the young boys like me to see someone they can identify with and know that they can do that too.*

Overall, experiences with the LGBTQ community were split: Some reported feeling a sense of belonging within the LGBTQ community, which had a significant and positive effect on their lives and health. But some participants had distant or conflicted relationships with the LGBTQ community and the resources offered. One participant described this duality in the following way: *They [the gay community] have a lot of knowledge. But*,* I feel there’s a lot of mean people and they can make fun of you and*,* you know*,* just like. You know how sometimes it can be some bitch*,* you know*,* queens and you know*,* they’re going to make you feel bad to make them feel better. Putting their expectations on you and stuff like that.*

#### Immigration as a contextual factor

As noted above, a substantial proportion of participants had recently migrated to the US from Latin America, making their way through Central America and Mexico to reach the US border, and had been living in the US for fewer than two years. A smaller proportion had immigrated from the Caribbean. Migration to the US was related to a range of factors. Some came to the US to avoid stigma or persecution related to their gender identity, sexual orientation, and/or HIV status. Another force driving immigration was hope for better employment and financial prospects. In many cases, they migrated to the US to obtain better HIV care and treatment, such as in cases where it was not possible to access HIV medications regularly in the home country. The US could offer hope of consistent treatment and a high quality of life, as Lionel, who spoke Spanish as their primary language, described:

*I’ve always dreamed*,* maybe because it’s in the United States that here people don’t care about your diagnosis*,* as long as you’re undetectable and you take your medication*,* people are going to accept you.*

But, the migration experience could disrupt HIV management. For example, Antonio, who spoke Spanish as their primary language, described how their medications were confiscated in Mexico but that they were hesitant to ask for them because of HIV-related stigma:

*So when I crossed the [Mexican] border*,* I had been imprisoned for eight days*,* but since I told the doctor in prison that I have HIV and I need medicine*,* I’m in bad shape. So that was when they decided to let me out…So because you were in a prison with many people*,* you get me? And I had my medicines…I had them in a bag. And they take those from you and they keep them for you until you leave from there*,* when they release you at the exit. So then to me*,* it gave me a lot of fear. The fear*,* being afraid to tell them that please*,* I needed that medicine. So then during the eight days I didn’t take it either.*

Some newcomer participants were applying for asylum, a complicated process that made securing HIV care in the US more difficult. Andres, who had moved to the US from central America, explained how their legal status negatively impacted access to housing, services, and employment and contributed to serious health issues, among other stressors:

*[Someone] broke into my room here in the shelter. They took my entire wallet with all my documents and now I only have copies of documents and now there is no way to fix it or where I should go to recover the lost documents. Unfortunately*,* [the HIV social services administration] has denied me support*,* telling me that I have to wait until my political asylum hearing. […] I’m being monitored for a stress crisis*,* anxiety*,* and problems. Because my dad died recently*,* and I haven’t been able to cope with my grief and I was really upset with that. That has given me a lot*,* a lot*,* a lot of depression. And apart from that*,* with the problem of migration*,* with the problem of [the HIV social services administration] that has not given me the support I need*,* is that I am without a job. So*,* all of that has given me a lot of pressure.*

These results highlight the challenges inherent in deciding to leave or needing to leave one’s country, particularly for younger people, and the resourcefulness they evidenced in their journeys and abilities to navigate the bureaucracies in the US. Despite participants’ desire to take HIV medication consistently, these contextual factors did not always allow them to do so. But, they were highly motivated to engage along the HIV care continuum as soon as they were able.

### Integration of quantitative and qualitative results

The interpretative community compared quantitative findings to qualitative findings, working domain by domain, and created a joint display table by consensus (Table 5). Because the qualitative in-depth interviews were semi-structured, we did not assume results from the qualitative effort would reflect every quantitative domain. We highlight a subset of the integrated findings presented in Table 5 in this section. Overall, the qualitative findings provided richness and context to quantitative results and help explain counter-intuitive results. We found with respect to housing and employment, qualitative results emphasized mainly unstable contexts and the mechanisms by which they affect HIV management, mainly in the past, compared to quantitative results which highlighted both protective and challenging contexts. Quantitative results yielded a negative association between receiving income from welfare, public assistance, etc. and being well-engaged in HIV care, but the qualitative data provided a rich and detailed description of the importance of government benefits for survival, along with the challenges participants faced managing large bureaucracies. Community resources were not assessed in the quantitative measure. Qualitative results provided a description of the reasons why participants did or did not engage with that community. Last, a number of factors were related to engagement in HIV care in the logistic regression, but did not predict HIV viral suppression at statistically significant levels. HIV care and viral suppression are strongly related, however. Yet in the qualitative data, relatively little was said about engagement in HIV care, and HIV medication adherence was an important and ever-present factor in their lives, whether they were currently taking HIV medication or not. Thus, in this case, quantitative and qualitative data were discrepant. In general, the two sets of results were complementary and qualitative results added detail and richness to the quantitative findings.

**Table 5:** Joint display summary to integrate and interpret findings

Quantitative finding	Qualitative finding	Comments and Interpretation
Both protective and challenging contexts were common. Challenging contexts experienced by some (approximately half the cohort) included temporary housing and unemployment. Most experienced financial hardship. These factors were not associated with engagement in care or HIV viral suppression, with the exception of income from federal benefits, as described below.	Challenging contexts were more commonly described than stable contexts. Factors that reduced stable housing including difficult family relationships, financial difficulties, and immigration (not having documentation/paperwork). These factors were described as having direct and indirect relationships to HIV viral suppression. HIV care was not typically seen as difficult to manage and relationships with providers were positive. Housing was assessed in the quantitative survey but the qualitative data highlighted that housing is a very prominent and ongoing challenge in participants’ lives, and critical to wellbeing. Participants were generally in a fairly stable place at the time of the interview but reflected on times of instability in the past.	Qualitative results emphasized mainly unstable contexts and the mechanisms by which they affect HIV management, mainly in the past, compared to quantitative results which highlighted both protective and challenging contexts.
Receiving income from welfare, public assistance, social security, disability, or workers compensation *negatively* related to engagement in HIV care, a counter-intuitive finding.	Benefits were generally seen as positive, particularly housing benefits. Participants often received their financial assistance and other benefits through a central NYC system which we refer to as the HIV social services administration. This large bureaucracy was challenging to navigate for these younger people. Immigrant participants who did not have proper paperwork had challenges getting benefits, and this created housing instability and financial precarity.	Quantitative and qualitative findings are discrepant. Receiving public benefits may be a proxy for extreme financial need, which can reduce engagement in HIV care.
Half of participants worked in the street economy.	Street economy involvement was driven by challenges finding work the formal economy, for example, because of transgender gender identity, or the inability to work because of a lack of documentation. Some participants sold or heard about others selling HIV medication, which can be considered a type of street economic activity that was not assessed in the quantitative measure.	Qualitative data uncovered the reasons why participants engage in the street economy, and added to the types of street economic activities (selling HIV medication).
Immigration: Half were born outside the US/Puerto Rico, only approximately half were US citizens, a third had refugee, asylum, or temporary protected immigrant status, and approximately a third spoke Spanish as their primary language. Descriptive data indicate 36% of those with HIV viral suppression spoke Spanish as their primary language compared to 19% among those not suppressed (Table 1) Those whose primary language was Spanish were more likely to be well-engaged in HIV care than English-speakers (Table 3)	Immigrant participants generally left their home countries to obtain better HIV care and/or avoid persecution for, primarily, their sexual orientation and gender identity, and secondarily, their HIV status. Immigrant participants faced challenges such as being unable to work in the formal economy or receive public assistance benefits and difficulties managing complex bureaucracies. Immigrant participants appeared to be coping well and managing life challenges, including HIV, with a constellation of personal resilience, support from family in the home country, and support from Latine-focused CBOs in the US.	Given the challenges immigrant participants face, including the lack of English proficiency among many, their successful HIV management is notable and warrants further study. These findings also suggest the challenges inherent in growing up in/living in the US as an AABL sexual and/or gender minority person living with HIV (e.g., structural racism, structural barriers to health, internalized stigma), compared to immigrants. More data on the length of time immigrant participants have lived in the US and their adaptation to the US are needed.
Number of adverse childhood experiences (ACES) negatively related to engagement in in HIV care but not HIV viral suppression.	We did not examine ACEs in the qualitative component of the present study in detail. In the transcripts, participants did not generally volunteer or describe experiences that reflect the 14 specific ACEs domains. However, experiences of discrimination and trauma were common. The type of trauma was generally not specified.	Quantitative data highlight the prevalence and importance of ACEs. More qualitative exploration is needed to understand the direct and indirect effects of specific ACEs and factors that buffer or exacerbate the negative effects of ACEs.
Community resources were not assessed.	Participants’ experiences with community resources, including LGBTQ community-based organizations, were mixed. Some found community-based organizations and/or the larger LGBTQ community supportive and others did not.	Qualitative findings addressed a gap in the quantitative assessment battery. The substantial lack of engagement in the LGBTQ community was an unexpected finding and may reflect changing norms in this age group.
A number of factors were related to engagement in HIV care in the logistic regression, but did not predict HIV viral suppression at statistically significant levels. HIV care and viral suppression are strongly related, however.	Participants had relatively little to say about engagement in HIV care, but HIV medication adherence was an important and ever-present factor in their lives, whether they were currently taking HIV medication or not.	It is possible that these background and contextual factors interact with variables at other levels of influence to predict HIV viral suppression. In the present study, the qualitative findings enhance quantitative results, even when discrepant, and point the way to new research questions.

## Discussion

The goal of the present study was to provide a rich description of sociodemographic and background domains and contextual factors among AABL young and emerging adults living with HIV. By recruiting from community settings, we captured a number of important but understudied subgroups in this larger population including those with non-suppressed HIV viral load, immigrant, refugee, and asylum-seeking individuals, monolingual Spanish-speaking persons, and those with serious socioeconomic disadvantage. These background factors complicate consistent engagement along the HIV care continuum. Our research with this cohort further highlights that this population also has barriers to research participation, even when they do occasionally or regularly present in clinical settings where they might possibly have the opportunity to engage in research [3]. For example, in this cohort, gaps of more than six months between HIV care appointments were common. Thus, the present study complements and extends research carried out in clinical settings and with persons consistently engaged along the care continuum. The mixed methods approach was useful in characterizing a number of background and contextual risk and protective factors and how they operate. The domains examined in the present study are largely distal to HIV management outcomes, but the study did advance what we know about AABL young and emerging adults living with HIV and aspects of HIV management.

On average, participants had been diagnosed with HIV four years prior, and as such, were relatively new to HIV management. Of note, economic hardship was the norm in this sample, which certainly complicates developmental processes and health behavior [54]. Participants were managing both expected and atypical developmental challenges, in the context of serious financial hardship. Similar to their peers not diagnosed with HIV, they grapple with education, work, finances, romantic and sexual relationships, and housing. But, they face additional challenges. All were AABL, almost all were sexual minorities (gay, bisexual, lesbian, queer, etc.), and about a third were non-cisgender (e.g., gender non-conforming, gender expansive). These identities, and their links to interconnected systems of oppression, complicate development, wellbeing, and functioning [55, 56]. In fact, sexual orientation and gender were the most frequent sources of discrimination in this sample. It was in this context that participants are managing living with HIV, a stigmatized and complex health condition. Notably, the majority of participants were well-engaged along the HIV care continuum at the time of study enrollment. The quantitative data identified some predictors of HIV care engagement, but not HIV viral suppression, as we describe throughout this section. The qualitative data provide a description of contextual factors that influence both HIV medication taking and HIV care engagement and how they operate.

Rates of adverse childhood experiences were marked in this sample. Generally, a score of four or more on an ACEs measure is considered significant and, in the general population, strongly related to adverse outcomes such as sexual risk taking, mental health, problematic drug use, and interpersonal and self-directed violence [57]. In our study, 80% scored four or higher on the ACEs scale. ACEs have received relatively little attention among young and emerging adults living with HIV, however. In a small study of young people living with HIV in the southern US, a third had an ACEs score of four or higher [58], substantially lower than in the present study. Among adults living with HIV, one study found the majority experienced one or more ACE (87%) [59]. In that study ACEs were not associated with the likelihood of HIV viral suppression, but ACEs were associated with poor health-related quality of life [59]. In this study, we found a relationship between higher ACEs scores and a lower odds of being well-engaged in HIV care, but, similar to this existing literature with adults, no relationship with HIV viral suppression. We interpret this finding as follows. First, most participants in the present study were well-engaged in HIV care and evidenced HIV viral suppression, despite high ACEs scores, on average, and various other risk factors. This suggests success on the part of HIV care and related social service systems, by and large, in engaging and treating AABL young and emerging adults living with HIV and also the current effectiveness and tolerability of HIV medications. It also suggests substantial resilience on the part of AABL young and emerging adults living with HIV, in that they overcome barriers and manage past traumatic experiences well enough to engage along the HIV care continuum. Further, we assume other protective factors not included in the present study are also at play. HIV care may be less important to the population compared to HIV medication, and therefore more vulnerable to the adverse effects of ACEs on health care engagement, either directly or indirectly. Further, this finding may reflect structural barriers the population experiences to HIV care engagement, such as transportation and scheduling difficulties. Overall, the present study, in combination with the existing literature, highlights the importance of addressing childhood traumas and their adverse effects in this population.

There is a large literature on how public (government) benefits such as financial assistance and the Supplemental Nutrition Assistance Program or “food stamps” program (SNAP) reduce or eliminate extreme poverty, food insecurity, unstable housing, and involvement in the street economy among persons living with HIV, thereby supporting HIV management [60–65]. In contrast to this literature, we found receiving income from welfare, public assistance, social security, disability, or workers’ compensation *reduced* the odds of being well-engaged in HIV care in the quantitative analysis. But, conversely, the qualitative results highlighted both the great importance of such benefits and their vital relationship to general stability and HIV management. However, participants faced difficulties accessing and maintaining benefits. Results highlight how challenging it can be for young and emerging adults to successfully interface with large and complex bureaucracies, such as the departments that administrate benefits and services to persons living with HIV. Immigrant participants, who often lack documentation and do not speak English, face additional barriers. Thus, we interpret the quantitative finding as indicating that the need for income support is a proxy for severe financial need, along with unemployment, and this severe need reduces engagement in HIV care, as has been found in past research [66]. In fact, in NYC, where the study was primarily located, the maximum amount of financial assistance provided is less than $400/month, the New York State-determined level of need [67]. It is likely that serious financial need may persist even when government income support is provided, as study qualitative findings clearly indicate. Moreover, one important finding in the present study is that involvement in the street economy (e.g., sex work, drug dealing, shoplifting) is common. Findings suggest that AABL young and emerging adults living with HIV who do not receive sufficient income assistance and are not employed in the formal economy engage in the street economy to survive. In particular, transgender/non-cisgender and immigrant individuals appear to have the greatest barriers to engagement in the formal economy and thus the greatest chances of engagement in the street economy, consistent with past research [68, 69]. Thus, increasing benefit income levels and providing training and support for employment have great potential to increase regular engagement in HIV care. Similarly, stable housing was vital for wellbeing and optimal HIV management, consistent with past research [70]. The present study extends past research by highlighting the importance of housing quality (e.g., privacy) and the need to match client needs and preferences with the specific type of housing.

The present study yields insights into the subset of the population characterized as immigrants, refugees, and asylum seekers. Among those in the sample, a third spoke Spanish as their primary or only language. But, despite challenges inherent in a lack of English fluency and, for many, lack of documentation, Spanish-speaking participants had a *higher* odds of being well-engaged in HIV care. In contrast, the existing literature generally finds that immigration status is a barrier to HIV care [71, 72]. Qualitative results shed light on why this might be the case. We found immigrant participants, among whom Spanish-speaking participants are a subgroup, generally left their home countries in large part to obtain better HIV care and/or avoid persecution for, primarily, their sexual orientation and gender identity, and secondarily, their HIV status. Their HIV management was supported by assistance from family in the home country and Latine-focused CBOs in the US, and personal resilience.

Moreover, immigrant persons may have different views on and experiences with HIV care compared to domestically born persons. For example, the literature suggests that medical mistrust is higher in domestically born compared to immigrant populations [73]. Study findings suggest that growing up in/living in the US as an AABL sexual and/or gender minority person living with HIV (e.g., where structural racism, structural barriers to health, internalized stigma are common) brings its own challenges, which immigrants do not experience. For example, our own past research examined the experience of non-immigrant low-income AABL older adult persons living with HIV over the long-term through the lens of symbolic violence. We found participants experienced a convergence of multiple social exclusions, harms, and stigmas, including structural racism and discrimination, which contributed to disengagement from HIV care and discontinuation of HIV medications. In particular, participants were “ground down” over time by material, social, and emotional challenges and this diminished self-worth and, at times, the will to live; social isolation and self-isolation, based in part on feeling devalued and dehumanized, served as stigma-avoidance strategies and mechanisms of social exclusion; and poor HIV management was internalized as a personal failure [74]. It is possible the non-immigrant AABL young and emerging adults living with HIV in the present study are similarly shaped by adverse structural and systemic factors in ways that immigrant persons are not.

### Limitations

The study has limitations. As noted above, the factors studied here are for the most part distal to HIV care and viral suppression, and may therefore interact with factors at other levels of influence, which we expect would limit the number and types of associations found. Further, as a mixed methods study, the qualitative research question was necessarily focused on specific domains, and not comprehensive. Our recruitment methods were not successful in reaching members of the population ages 16–18 years, which suggests that different strategies are needed to reach and enroll the youngest people in this population. Similarly, those with non-suppressed HIV viral load, an important focus of the larger study, were difficult to locate and engage. National data cited above suggest that at least half of the population does not sustain HIV viral suppression, but only 19% of the present study had viral non-suppression. There is diversity among the samples of immigrant participants with respect to country of origin, reasons for migrating, and length of time in the US that we did not explore here related to the need to keep the analysis focused. Last, NYC and Newark, NJ are relatively service-rich environments compared to other geographical locations. These issues limit inferences that can be made from the present study.

### Implications and recommendations

In Table 6, we present implications and recommendations drawn from the present study. We summarize them briefly here. One set of recommendations focuses on the importance of maintaining or enhancing services for this population to support consistent engagement along the HIV care continuum. This includes services appropriate for the emerging adult developmental period, those whose primary language is Spanish, and immigrants, and resources to address structural barriers. Further, since those in this population who identify as LGBTQ are diverse, a range of services is needed. Further, adverse childhood experiences are a serious issue in this population and warrant prevention and mitigation. Last, community-based research for this population is needed to complement studies in medical settings.

**Table 6:** Implications and recommendations from the present study

Domain	Implication or recommendation
Developmental period	Since it is not typical for emerging adults to manage chronic and stigmatized health conditions along with other developmental challenges, developmentally appropriate resources, services, and supports are vital
Structural barriers	Structural barriers impede consistent engagement along the HIV care continuum (unstable housing, unemployment, poverty) and financial benefits and support services are needed to overcome them
Language and immigration	Culturally responsive services are needed, for example, services in one’s primary language as well as immigration support and legal services
Benefits and services	Federal benefits and the local HIV social services administration are vital and warrant expansion. Since benefit and service levels can wax and wane, research is needed to monitor policy changes and their effects, and advocacy groups may need to consider alternative sources of financial supports and social services when supports are lacking.
Adverse childhood experiences	Given the high rates of adverse childhood experiences in this population and its negative effects on engagement in HIV care, trauma-informed care is essential, along with efforts to prevent and mitigate such experiences
LGBTQ community	Opinions regarding the utility of the LGBTQ community and its services were mixed. Since AABL emerging adults living with HIV are diverse, including among those who identify as sexual and gender minority individuals, a range of programming and services is required
Spanish-speaking participants	Counter-intuitive findings, such as Spanish-speaking participants being more likely to be well-engaged in HIV care than English-speaking persons (most born in the US), suggest challenges inherent in living as an AABL person in the US, which can be studied and mitigated, along with the need to understand resilience
Community-based research	Community-based research for this population is needed to complement studies in medical settings, since barriers to consistent engagement in HIV care are serious and past lapses in care are common

## Conclusions

The population of AABL younger persons living with HIV will change over time. Studies carried out in community settings are an important complement to those in clinical venues, including to capture new and understudied subpopulations and those with barriers to HIV care settings and research participation. The mixed methods approach was useful and the present study advances what is known about socio-demographic, background, and contextual factors among a diverse sample of AABL young and emerging adults living with HIV, including counter-intuitive findings, substantial resilience, and new possible areas of exploration.

## Electronic supplementary material

Below is the link to the electronic supplementary material.

Supplementary Material 1

Supplementary Material 2

Supplementary Material 3

## Data Availability

Data are available upon reasonable request from the corresponding author.

## Abbreviations

AABL: African American/Black or Latine
ACEs: Adverse Childhood Experiences
CBO: Community-based organizations
CDC: Centers for Disease Control and Prevention
LGBTQ: Lesbian, gay, bisexual, transgender, and queer
NJCRI: North Jersey Community Research Initiative
NYC: New York City
OR: Odds ratio
SRO: Single room occupancy (residence)
US: United States
