# Supplementary material for: A mixed methods descriptive study of a diverse cohort of African American/Black and Latine young and emerging adults living with HIV: Sociodemographic, background, and contextual factors
Source: BMC Public Health. 2025 Feb 14;25:620. doi: 10.1186/s12889-025-21869-3 (PMC11829469; doi:10.1186/s12889-025-21869-3)
Supplement: Supplementary file 1 — Supplementary Material 1 [file 12889_2025_21869_MOESM1_ESM.docx]

| **Supplemental Table 1: Sociodemographic and background characteristics [%, (N)]** | | | |
| --- | --- | --- | --- |
|  | **Overall (N=271)** | **Suppressed (N=219)** | **Not Suppressed (N=52)** |
| *Gender identity* |  |  |  |
| Man, male | 63.1 (171) | 62.6 (137) | 65.4 (34) |
| Gender non-binary | 12.2 (33) | 11.9 (26) | 13.5 (7) |
| Transfemale/Transgender Woman | 6.6 (18) | 7.3 (16) | 3.8 (2) |
| Woman, female | 5.5 (15) | 4.6 (10) | 9.6 (5) |
| Transgender | 4.1 (11) | 4.6 (10) | 1.9 (1) |
| More Than One Identity | 3.0 (8) | 3.7 (8) | 0 (0) |
| Genderfluid | 1.8 (5) | 0.9 (2) | 5.8 (3) |
| Genderqueer | 1.8 (5) | 2.3 (5) | 0 (0) |
| None of these describe me, other | 0.7 (2) | 0.9 (2) | 0 (0) |
| Prefer not to answer | 0.4 (1) | 0.5 (1) | 0 (0) |
| Questioning, or unsure of your gender identity | 0.4 (1) | 0.5 (1) | 0 (0) |
| Transman/ Transgender Man | 0.4 (1) | 0.5 (1) | 0 (0) |
|  |  |  |  |
| *Sexual orientation* |  |  |  |
| Gay | 60.9 (165) | 63.5 (139) | 50.0 (26) |
| Bisexual | 14.4 (39) | 12.8 (28) | 21.2 (11) |
| Pansexual | 9.2 (25) | 9.6 (21) | 7.7 (4) |
| Heterosexual or straight | 6.6 (18) | 6.4 (14) | 7.7 (4) |
| Prefer not to answer | 3.3 (9) | 3.2 (7) | 3.8 (2) |
| Queer | 1.8 (5) | 1.4 (3) | 3.8 (2) |
| Other | 1.1 (3) | 0.9 (2) | 1.9 (1) |
| More Than One Orientation | 1.1 (3) | 0.9 (2) | 1.9 (1) |
| Do not use labels to identify yourself | 0.7 (2) | 0.9 (2) | 0 (0) |
| Lesbian | 0.4 (1) | 0.5 (1) | 0 (0) |
| Two-spirit | 0.4 (1) | 0 (0) | 1.9 (1) |
|  |  |  |  |
| *Primary recruitment sources* |  |  |  |
| Peer recruitment | 50.2 (136) | 51.6 (113) | 44.2 (23) |
| Dating apps (Grindr, Jack'd, etc.) | 23.6 (64) | 22.8 (50) | 26.9 (14) |
| Saw post in Craigslist | 5.5 (15) | 5.0 (11) | 7.7 (4) |
|  |  |  |  |
| *Immigration and citizenship status* |  |  |  |
| US citizen | 53.5 (145) | 50.7 (111) | 65.4 (34) |
| Refugee, asylum, temporary protected  immigrant status | 35.1 (95) | 37.9 (83) | 23.1 (12) |
| Undocumented | 6.6 (18) | 6.4 (14) | 7.7 (4) |
| Permanent resident/ Green card | 3.7 (10) | 3.7 (8) | 3.8 (2) |
| Valid tourist visa/ Work visa or permit/ student  visa | 0.7 (2) | 0.9 (2) | 0 (0) |
|  |  |  |  |
| Resides in NJ | 8.5 (23) | 9.1 (20) | 5.8 (3) |
