# Supplementary material for: A mixed methods descriptive study of a diverse cohort of African American/Black and Latine young and emerging adults living with HIV: Sociodemographic, background, and contextual factors
Source: BMC Public Health. 2025 Feb 14;25:620. doi: 10.1186/s12889-025-21869-3 (PMC11829469; doi:10.1186/s12889-025-21869-3)
Supplement: Supplementary file 2 — Supplementary Material 2 [file 12889_2025_21869_MOESM2_ESM.docx]

| **Supplemental Table 2. HIV history, HIV care engagement, and Health (M, [SD] or %, N)** | | | |
| --- | --- | --- | --- |
|  | **Overall (N=271)** | **Suppressed (N=219)** | **Not Suppressed (N=52)** |
| Started HIV medication 6 months or longer after first diagnosis | 13.3 (35) | 11.8 (25) | 19.6 (10) |
|  |  |  |  |
| Longest duration of taking HIV medication, in months | 22.0 (68.6) | 23.2 (75.8) | 17.0 (18.0) |
| Median [Q1, Q3] | 12.0 [6.00, 36.0] | 12.0 [6.75, 43.5] | 12.0 [3.50, 24.0] |
|  |  |  |  |
| Number of times stopped taking HIV medication for at least two days, then started again | 7.31 (28.8) | 6.31 (29.8) | 11.5 (24.1) |
| Median [Q1, Q3] | 1.00 [0, 4.00] | 1.00 [0, 3.25] | 3.00 [1.00, 7.00] |
|  |  |  |  |
| Ever has gone 6 months or longer without a medical visit with HIV care provider | 33.9 (92) | 26.9 (59) | 63.5 (33) |
|  |  |  |  |
| Received any HIV care in the past year | 96.3 (261) | 98.6 (216) | 86.5 (45) |
|  |  |  |  |
| Fully vaccinated for COVID-19 (primary series) | 88.9 (241) | 91.8 (201) | 76.9 (40) |
|  |  |  |  |
| *STI diagnosis in the past year* |  |  |  |
| Chlamydia | 21.0 (55) | 21.3 (45) | 19.6 (10) |
| Genital warts, anal warts, human  papillomavirus (HPV) | 7.0 (18) | 7.2 (15) | 5.9 (3) |
| Gonorrhea | 24.0 (63) | 25.9 (55) | 16.0 (8) |
| Herpes, HSV1 or HSV2 | 3.8 (10) | 4.2 (9) | 2.0 (1) |
| Syphilis | 32.4 (84) | 35.4 (74) | 20.0 (10) |
|  |  |  |  |
| Ever heard of pre-exposure prophylaxis (PrEP) | 74.2 (201) | 74.4 (163) | 73.1 (38) |
| Ever took pre-exposure prophylaxis (PrEP) | 15.9 (43) |  | 7.7 (4) |
