# Supplementary material for: A mixed methods descriptive study of a diverse cohort of African American/Black and Latine young and emerging adults living with HIV: Sociodemographic, background, and contextual factors
Source: BMC Public Health. 2025 Feb 14;25:620. doi: 10.1186/s12889-025-21869-3 (PMC11829469; doi:10.1186/s12889-025-21869-3)
Supplement: Supplementary file 3 — Supplementary Material 3 [file 12889_2025_21869_MOESM3_ESM.docx]

| **Supplemental Table 3. Action Contexts (%, N)** | | | | | |
| --- | --- | --- | --- | --- | --- |
|  | **Overall (N=271)** | **Suppressed (N=219)** | | **Not Suppressed (N=52)** | |
| *Current living arrangements* |  |  | |  | |
| Your own house or apartment | 44.3 (120) | 42.9 (94) | | 50.0 (26) | |
| In a shelter, single-room occupancy hotel or some other residential facility (halfway house, drug treatment) | 37.6 (102) | 37.0 (81) | | 40.4 (21) | |
| Your own room in your parent(s) or another family member's house or apartment | 8.9 (24) | 10.5 (23) | | 1.9 (1) | |
| A shared room or couch in your parent(s) or another family member's house or at someone else's house or apartment | 6.3 (17) | 6.4 (14) | | 5.8 (3) | |
| Other | 1.8 (5) | 2.3 (5) | | 0 (0) | |
| Some other place not intended for habitation including on the street, vacant lot, abandoned building, park | 1.1 (3) | 0.9 (2) | | 1.9 (1) | |
|  |  |  | |  | |
| *Developmental milestones* |  |  | |  | |
| *Relationship status* |  |  | |  | |
| Single, not seeking a sexual or romantic partner or partners | 36.5 (99) | 36.5 (80) | | 36.5 (19) | |
| Single, seeking a sexual or romantic partner or partners | 26.9 (73) | 26.5 (58) | | 28.8 (15) | |
| Dating or "seeing" one or more persons | 17.3 (47) | 17.4 (38) | | 17.3 (9) | |
| Domestic partnership/living with partner | 8.9 (24) | 9.6 (21) | | 5.8 (3) | |
| In a long-term relationship | 8.9 (24) | 9.6 (21) | | 5.8 (3) | |
| Married | 4.4 (12) | 4.1 (9) | | 5.8 (3) | |
|  |  |  | |  | |
| Had sexual intercourse over the lifetime | 93.4 (253) | 92.7 (203) | | 96.2 (50) | |
|  |  |  | |  | |
| Currently attending school or an academic  program | 17.7 (48) | | 19.6 (43) | | 9.6 (5) |
|  |  | |  | |  |
| *Current employment status* |  | |  | |  |
| Not working but actively looking | 52.8 (143) | | 51.1 (112) | | 59.6 (31) |
| Working part-time | 20.7 (56) | | 23.7 (52) | | 7.7 (4) |
| Working full-time | 12.9 (35) | | 11.9 (26) | | 17.3 (9) |
| Not working but not actively looking | 7.7 (21) | | 8.2 (18) | | 5.8 (3) |
| Working off-the-books or in the street economy (panhandling, recycling, selling drugs or sex) | 4.1 (11) | | 3.7 (8) | | 5.8 (3) |
|  |  | |  | |  |
| *Street economy activities - lifetime* |  | |  | |  |
| Traded sex for money, drugs, food, or a place to stay | 41.0 (111) | | 38.4 (84) | | 51.9 (27) |
| Stolen or shoplifted something from a store | 20.7 (56) | | 17.4 (38) | | 34.6 (18) |
| Been involved in dealing, selling, bagging, or running drugs | 15.1 (41) | | 12.3 (27) | | 26.9 (14) |
| Panhandled, or asked for spare change | 7.7 (21) | | 6.8 (15) | | 11.5 (6) |
| Broken into a house, store, or car when no one was there | 4.4 (12) | | 2.7 (6) | | 11.5 (6) |
| Pimped someone | 4.4 (12) | | 2.7 (6) | | 11.5 (6) |
| Mugged or robbed someone | 3.0 (8) | | 1.8 (4) | | 7.7 (4) |
| Anything else in the street economy | 6.6 (18) | | 6.8 (15) | | 5.8 (3) |
|  |  | |  | |  |
|  |  | |  | |  |
